# High frequency of the risk allele of rs4132601 and rs11978267 from the *IKZF1* gene in indigenous Mexican population

**DOI:** 10.1002/mgg3.1589

**Published:** 2021-01-16

**Authors:** Jorge Gutiérrez‐Franco, Miriam Fabiola Ayón‐Pérez, Ma. de Jesús Durán‐Avelar, Norberto Vibanco‐Pérez, Diego Eduardo Sánchez‐Jasso, Dulce Guadalupe Bañuelos‐Aguayo, Jaime Sánchez‐Meza, Helia Judith Pimentel‐Gutiérrez, José Francisco Zambrano‐Zaragoza, Juan Manuel Agraz‐Cibrián, Alejandro Vázquez‐Reyes

**Affiliations:** ^1^ Laboratorio de Inmunología Unidad Académica de Ciencias Químico‐Biológicas y Farmacéuticas Universidad Autónoma de Nayarit Tepic Nayarit México; ^2^ Laboratorios de Investigación en Biología Molecular e Inmunología Unidad Académica de Ciencias Químico Biológicas y Farmacéuticas Universidad Autónoma de Nayarit Tepic Nayarit México; ^3^ Charité Universitätsmedizin, Virchow‐Klinikum Berlin Germany

**Keywords:** ALL, genetic polymorphism, *IKZF1*, Indigenous Mexican

## Abstract

**Background:**

*IKZF1* is a relevant gene associated with the pathogenesis of acute lymphoblastic leukemia, and the *rs*4132601 (T>G) and *rs*11978267 (A>G) polymorphisms have been associated with the development of this disease in several populations. The aim of this study was to determine the allelic and genotypic frequencies of the *rs*4132601 and *rs*11978267 polymorphisms in two indigenous Mexican groups (Cora and Huichol) and Mestizo populations from Nayarit, Mexico, and compare them with the frequencies of both polymorphisms in other populations of the world.

**Methods:**

One hundred, 116, and 100 subjects from the Mestizo, Huichol, and Cora populations, respectively, all of them residents of the state of Nayarit, Mexico, were analyzed. The frequencies of rs4132601 and rs11978267 were determined by allelic discrimination using TaqMan assays.

**Results:**

The allelic frequencies of rs4132601 were as follows: Mestizo group T = 0.74, G = 0.26; Cora T = 0.745, G = 0.255; and Huichol T = 0.47, G = 0.53. In the case of the rs11978267 polymorphism, the allelic frequencies were Mestizo A = 0.745, G = 0.255; Cora A = 0.735, G = 0.265; and Huichol A = 0.457, G = 0.543. For each population, both polymorphisms were in Hardy–Weinberg equilibrium.

**Conclusion:**

The Huichol population from Nayarit presented the highest frequencies of the risk allele reported to date in the whole world for both *rs*4132601 and *rs*11978267 polymorphisms.

## Graphical abstract

An indigenous Mexican population presented the highest frequencies of the risk allele reported to date in the whole world for both rs4132601 and rs11978267 polymorphisms. The number of individuals analyzed from this population, represents the 1% of the total population from Nayarit.

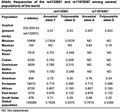

## 1 INTRODUCTION

**FIGURE 1:**
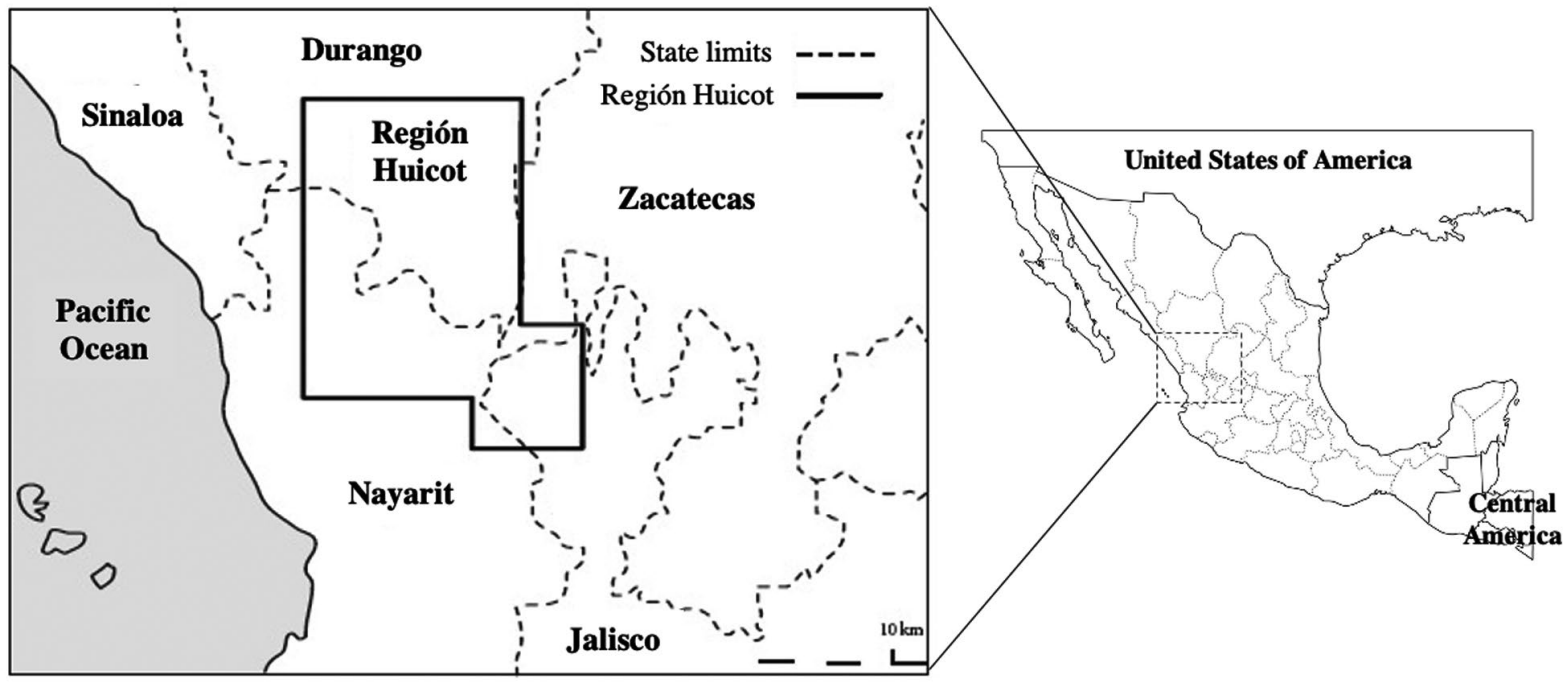
Map of the “Huicot Region or El Gran Nayar”. It is a cultural region where four indigenous Mexican groups live: Huichol, Cora, Tepehuanos and Nahuas or Mexicaneros. The region covers the mountain of the states of Nayarit, Jalisco, Durango and Zacatecas (Guízar Vázquez, 2005)

*IKZF1* (IKAROS Family Zinc Finger 1) (OMIM 603023) is considered a relevant gene associated with acute lymphoblastic leukemia (ALL), which is located on 7p12.2 and contains eight exons. *IKZF1* encodes the protein IKAROS, an anti‐leukemic transcriptional factor that is highly conserved and essential for the differentiation of the B lineage in hematopoietic stem cells and as a tumor suppressor in B‐cell ALL (Yokota & Kanakura, 2016). The loss or functional failure of *IKZF1* can be mediated by several molecular mechanisms, such as alterations in the copy number of the *IKZF1* locus, single‐nucleotide polymorphisms (SNPs), and partial or complete gene deletions producing haploinsufficiency (Davis, 2011). Approximately 15%–20% of children diagnosed with B‐cell precursor ALL (BCP‐ALL) show deletions in *IKZF1*; this figure increases to ~70% in Philadelphia‐chromosome‐positive (Ph+) and ~40% in Ph‐like‐chromosome‐positive individuals (Boer et al., 2016). In BCP‐ALL, deletions and mutations in *IKZF1* are significantly associated with increased risk and relapse (Rogers et al., 2018). Significant associations have been reported between BCP‐ALL and the SNPs rs4132601T>G, rs11978267 A>G, rs11980379T>C, and rs10272724T>C in *IKZF1* (Bahari et al., 2016; Dai et al., 2014; Xu et al., 2013). The polymorphisms *rs*11978267 and *rs*4132601 have been associated with BCP‐ALL (both present an OR = 1.69 (1.58–1.81)) in European, European‐American, Afro‐American, and Hispano‐American populations. Specifically, both polymorphisms increase the risk of BCP‐ALL in European populations (Dai et al., 2014). However, several studies have reported controversial results in Asian and African populations (Dai et al., 2014; Emerenciano et al., 2014; Lin et al., 2014), probably because the genetic variability and heterogeneity in populations play an important role. The variability of the Mexican population and ethnic groups from region to region may influence the genetic distribution. Mexico has approximately 124 million people (INEGI, 2017), and around 12 million people belong to 62 identified ethnic groups. Cora is an indigenous group of 15,994 individuals, living in the municipality of El Nayar, at the Sierra of Nayarit, in the west of the State of Nayarit (15,811 individuals), also known as the “Huicot Region or El Gran Nayar” (Figure 1), and others live in the State of Durango (183 individuals) (Dahlgren de Jordan, 1994; SIC, 2019a). Huichol is an indigenous group with a total population of 23,769 individuals, that lives at the Sierra Madre Occidental, in the states of Nayarit (11,978 individuals), Jalisco (10,305 individuals), and Durango (1486 individuals) (Jáuregui & Neurath, 2003; SIC, 2019b). The Mestizo population from Mexico presents an admixture of European, African, and indigenous genes (Galanter et al., 2012; Jimenez‐Sanchez et al., 2008). Hence, the analysis of genomic polymorphisms in ethnic groups may contribute significant information on this topic. Some reports show the frequencies of polymorphisms rs4132601 and rs11978267 in populations of Mexican origin (HapMap, 2019; PAGE‐study, 2019), but there are no studies in indigenous Mexican groups, who live in an isolated manner, reproducing with other members of their community and maintaining their genetic features (Dahlgren de Jordan, 1994). The aim of the study was to determine the allelic and genotypic frequencies of the rs4132601 and rs11978267 polymorphisms in indigenous Mexican groups (Cora and Huichol) and the Mestizo population from Nayarit, Mexico and compare them with the frequencies in other populations in the world.

## 2 MATERIALS AND METHODS

### 2.1 Study groups

Male and female Mestizos >18 years old from Nayarit and the Amerindian populations from the region of “Huicot Region or El Gran Nayar,” Nayarit were included. DNA samples were taken from 316 selected subjects: 100 subjects from the Mestizo population (*n* = 200 alleles), 100 from the Cora population (*n* = 200 alleles) for both polymorphisms, 116 from the Huichol population (*n* = 232 alleles) for rs11978267 and 100 from the Huichol population (*n* = 200 alleles) for rs4132601. Mestizo subjects were defined as those born in Nayarit, Mexico, who spoke Spanish, had Mexican parents, and did not belong to any native group. The indigenous Mexican subjects spoke their native language and had three generations belonging to the ethnic group. All subjects received an explanation of the features of the study prior to inclusion.

### 2.2 Ethical compliance

Informed consent was obtained from all individual participants included in the study. The study was conducted according to the ethical standards of the World Medical Association (Declaration of Helsinki) (Association, 2014). The project was approved by the Secretaría de Investigación y Posgrado from the Universidad Autónoma de Nayarit, number SIP‐18077.

### 2.3 DNA extraction

DNA was extracted from peripheral blood using the QIAamp DNA Blood Mini Kit (QIAGEN^®^), according to the manufacturer's guidelines. Samples were quantified using an Eppendorf BioPhotometer^®^ D30 spectrophotometer, and the DNA concentration was established at 2.5 ng/μl for PCR analysis. Samples were stored at –80°C until genotyping analysis.

### 2.4 Genotyping

Genotyping of the rs4132601 and rs11978267 polymorphisms was carried out by Real‐Time PCR, using predesigned TaqMan^®^ SNP Genotyping Assays: C__26019772_10 and C_199413_10 (Applied Biosystems™), respectively. Briefly, the PCR reactions were carried out in a total volume of 10 µL, 5 µL of TaqMan™ Genotyping Master Mix (Applied Biosystems™), 0.25 µl of the predesigned TaqMan^®^ SNP Genotyping Assay (C__26019772_10 or C_199413_10), 1 µl of DNA sample (2.5 ng/µl), and 3.75 µl of nuclease‐free water. The reactions were carried out in an ABI 7500 Fast Real‐Time thermocycler using the standard conditions recommended by the manufacturer: 95°C for 10 minutes and 40 cycles of 95°C for 15 seconds and 60°C for 1 minute. The data were analyzed by means of an allelic discrimination assay using the SDS software (Applied Biosystems™). The samples were analyzed out in duplicate, and positive and negative controls were used in each genotyping analysis.

### 2.5 Statistical analysis

To compare the genotypic and allelic frequencies in Mestizo, Cora, and Huichol populations, the Minitab 17 software was used. The allelic and genotypic frequencies from Mestizo versus Cora, Mestizo versus Huichol, and Huichol versus Cora were compared. Also, the allelic frequencies from Mestizo, Cora, and Huichol populations were compared with the global allelic frequency. The SNPs analyzed were tested for Hardy–Weinberg equilibrium in the Mestizo, Cora, and Huichol populations. A confidence level of 95% was used and *p* < 0.05 was considered as statistically significant.

## 3 RESULTS

### 3.1 Distribution of the allelic and genotypic frequencies of rs4132601 and rs11978267 polymorphisms

The three populations analyzed were in Hardy–Weinberg Equilibrium (HWE) (*p* > .05). There was linkage disequilibrium (LD) between the two polymorphisms evaluated in IKZF1 gene for Cora (*D*′ = 0.2407, *r* ^2^ = .0572, *p* < .001) and Huichol population (*D*′ = 0.2799, *r* ^2^ = .0736, *p* < .001). The distribution of the *IKZF1* rs4132601 and rs11978267 polymorphisms among the three populations is shown in Table 1 and the haplotype frequencies estimation for Cora and Huichol populations are shown in Table 2.

**TABLE 1:** Allelic and genotypic frequency of the polymorphisms in the Mestizo and indigenous Mexican populations

Variant	Mestizo	Cora	Huichol
rs4132601			
n	100	100	100
TT	54 (0.54)	53 (0.53)	24 (0.24)
TG	40 (0.4)	43 (0.43)	46 (0.46)
GG	6 (0.06)	4 (0.04)	30 (0.3)
T	148 (0.74)	149 (0.745)	96 (0.47)
G	52 (0.26)	52 (0.255)	104 (0.53)
HWE	0.43	0.29	0.8
rs11978267			
n	100	100	116
AA	57 (0.57)	52 (0.52)	27 (0.23)
AG	35 (0.35)	43 (0.43)	52 (0.45)
GG	8 (0.08)	5 (0.05)	37 (0.32)
A	149 (0.745)	147 (0.735)	106 (0.457)
G	51 (0.255)	53 (0.265)	126 (0.543)
HWE	0.43	0.44	0.35

Note

n, number of subjects analyzed. Allelic frequencies 2n (frequency). Genotypic frequency n (frequency).

Abbreviation: HWE, Hardy–Weinberg Equilibrium.

**TABLE 2:** Haplotype frequencies of the polymorphisms in the Mestizo, Cora, and Huichol populations

Mestizo vs. Cora (*n* = 200)	rs4132601	rs11978267	Total	Mestizo	Cora	Cumulative frequency
1	T	A	0.5953	0.5384	0.6689	0.5953
2	G	G	0.1128	0.0534	0.1889	1
3	T	G	0.1472	0.2016	0.0761	0.7425
4	G	A	0.1447	0.2066	0.0661	0.8872

Mestizo vs. Huichol (*n* = 216)	rs4132601	rs11978267	Total	Mestizo	Huichol	Cumulative frequency
1	T	A	0.4193	0.5384	0.3111	0.4193
2	G	G	0.23	0.0534	0.3936	0.6493
3	T	G	0.1797	0.2016	0.1495	0.829
4	G	A	0.171	0.2066	0.1458	1

Abbreviation: n, number of subjects analyzed.

### 3.2 Genotypic and allelic frequencies of the rs4132601 and rs11978267 polymorphisms in Mestizo, Cora, and Huichol populations

A two‐proportions test for statistically significant differences between allelic and genotypic frequencies was conducted. Table 3 shows a comparison of frequencies between Mestizo versus Cora populations, Mestizo versus Huichol populations, and Cora versus Huichol populations. We found statistically significant differences in the allelic frequencies of Huichol versus Mestizo and Huichol versus Cora populations in alleles T and G (rs4132601), as well as A and G (rs11978267). Furthermore, significant differences were found in the frequencies of genotypes TT and GG (rs4132601), as well as AA and AG (rs11978267), of the Huichol versus Mestizo and Huichol versus Cora populations (Table 3).

**TABLE 3:** Comparison of the allelic and genotypic frequencies of the polymorphisms among the populations analyzed

Popu**lation**	**Genotype**			**Allele**	
	TT	TG	GG	T	G
**rs4132601**					
Mestizo vs. Cora	0.54 vs. 0.53	0.4 vs. 0.43	0.06 vs. 0.04	0.74 vs. 0.745	0.26 vs. 0.255
Mestizo vs. Huichol	*	0.4 vs. 0.46	*	*	*
Cora vs. Huichol	*	0.43 vs. 0.46	*	*	*
	AA	AG	GG	A	G
rs11978267					
Mestizo vs. Cora	0.57 vs. 0.52	0.35 vs. 0.43	0.08 vs. 0.05	0.745 vs. 0.735	0.255 vs. 0.265
Mestizo vs. Huichol	0.57 vs. 0.23*	0.35 vs. 0.45	0.08 vs. 0.32*	0.745 vs. 0.457*	0.255 vs. 0.543*
Cora vs. Huichol	0.52 vs. 0.23*	0.43 vs. 0.45	0.05 vs. 0.32*	0.735 vs. 0.457*	0.265 vs. 0.543*

* *p* < 0.05.

### 3.3 Comparison of the frequencies of the rs4132601 (ClinVar accession number SCV000993544) and rs11978267 (ClinVar accession number SCV000993545) polymorphisms among populations of the world

The mainly frequencies of the rs4132601 and the rs11978267 of the populations of the world are shown in Table 4. In Mestizo and Cora populations, the frequencies of the ancestral allele, T (rs4132601) and A (rs11978267) alleles, were the highest. Moreover, in the Huichol population, the frequency of the risk allele, G allele, of both polymorphisms was the highest, even higher than those reported in other populations of the world (Table 4).

**TABLE 4:** Allelic frequencies of the rs4132601 (ClinVar accession number: SCV000993544) and rs11978267 (ClinVar accession number: SCV000993545) polymorphisms among several populations of the world reported on the NIH

Population	Group	Sample size (alleles)	rs4132601		rs11978267		Reference
			Ancestral allele T	Polym allele G	Ancestral allele A	Polym allele G	
Mexican	Study‐wide	632 (600 for rs4132601)	0.655	0.345	0.636	0.364	Present study
	Mestizo	200	0.74	0.26	0.745	0.255	
	Cora	200	0.745	0.255	0.735	0.265	
	Huichol	232 (200 for rs4132601)	0.47	0.53	0.457	0.543	
Mexican	NS	10,806	0.7424	0.2576	ND	ND	PAGE‐study (2019)
Puerto Rican	NS	7918	0.751	0.249	ND	ND	PAGE‐study (2019)
Cuban	NS	4230	0.742	0.258	ND	ND	PAGE‐study (2019)
Dominican	NS	3828	0.785	0.215	ND	ND	PAGE‐study (2019)
Native American	NS	1260	0.752	0.248	ND	ND	PAGE‐study (2019)
Mexican	NS	100	ND	ND	0.84	0.16	HapMap (2019)
European^a^	NS	18,472	0.715	0.285	0.712	0.288	gnomAD‐Study (2019a, 2019b)
African	NS	8728	0.807	0.193	0.807	0.193	gnomAD‐Study (2019a, 2019b)
East Asian	NS	1616	0.878	0.122	0.878	0.122	gnomAD‐Study (2019a, 2019b)
American	NS	836	0.75	0.25	0.76	0.24	gnomAD‐Study (2019a, 2019b)
South Asian	NS	978	0.71	0.29	0.68	0.32	1000Genomes‐study (2019a, 2019b)
Estonian	Study‐wide	4480	0.707	0.293	0.703	0.297	Stonian‐population‐study (2019a, 2019b)
Global population	Study‐wide	125,568	0.7628	0.2372	0.7615	0.2385	TopMed‐study (2019a, 2019b)

Abbreviations: ND, Not determined; NS, Not specified; Polym, Polymorphic.

^a^ All European population (includes Finnish population).

### 3.4 Allelic frequencies of Mestizo, Cora, and Huichol populations versus the global population

The allelic frequencies obtained in the present study were compared with those reported in the global population (Table 4) (TopMed‐study, 2019a, 2019b). In the Huichol population, the frequencies of the T = 0.47 (rs4132601) and A = 0.457 (rs11978267) alleles were statistically lower (*p* < .001), while the frequencies of the G (0.53) and G (0.543) alleles, respectively, were statistically higher (*p* < .001), when compared with the frequencies reported in the global population (TopMed‐study, 2019a, 2019b) (Table 5). Moreover, the allelic frequencies of the Mestizo and Cora populations did not show statistically significant differences when compared with the allelic frequencies reported in the global population (Table 5).

**TABLE 5:** Comparison among the allelic frequencies of the Mestizo, Cora, and Huichol populations versus Global frequencies

Population	rs4132601		rs11978267	
	T	G	A	G
Allelic frequencies of the global population^a^ vs.				
Huichol	*p* < .001	*p* < .001	*p* < .001	*p* < .001
Cora	*p* = .560	*p* = .560	*p* = .397	*p* = .35
Mestizo	*p* = .459	*p* = .459	*p* = .593	*p* = .534

^a^ Allelic frequencies reported by TOPMed (TopMed‐study, 2019a, 2019b). Statistical analysis from the comparison of the allelic frequencies of Global, Mestizo, Cora, and Huichol populations by a two proportion *z*‐test.

### 3.5 Frequencies in Mestizo, Cora, and Huichol populations versus previous reports from Mexican population

The allelic frequencies of the rs4132601 and rs11978267 in Mestizo, Cora, and Huichol populations were compared against previously reported frequencies in the Mexican population (HapMap, 2019; PAGE‐study, 2019) (Table 4). A comparison of these frequencies is shown in Table 6.

**TABLE 6:** Comparison among the allelic frequencies of the Mestizo, Cora, and Huichol populations with the allelic frequencies of Mexican populations

Population	rs4132601		rs11978267	
	T	G	A	G
Frequency in MXN^a^	0.7424	0.2576	0.840	0.16
Frequency in Mestizo	0.74	0.26	0.745	0.255
MXN vs. Mestizo	*p* = .94	*p* = .94	*p* = .025	*p* = .013
Frequency of Cora	0.745	0.255	0.735	0.265
MXN vs. Cora	*p* = .933	*p* = .93	*p* = .013	*p* = .013
Frequency in Huichol	0.47	0.53	0.457	0.543
MXN vs. Huichol	*p* < .001	*p* < .001	*p* < .001	*p* < .001

^a^ Allelic frequencies of a Mexican population (MXN), reported by (PAGE‐study, 2019) for rs4132601 and (HapMap, 2019) for rs11978267.

## 4 DISCUSSION

ALL has a multifactorial origin in which genetic variations such as translocations, deletions, insertions, or SNPs play an important role, for instance, in response to xenobiotics, in immune system regulation, DNA repair mechanisms, and gene regulation by transcriptional factors that predispose to the development of ALL (Jiménez‐Morales et al., 2017). These molecular markers associated with the development of ALL demonstrate genetic variability between different ethnic groups. The Hispanic population is more susceptible to developing ALL compared to non‐Hispanics (McNeil et al., 2002). In Mexico, between 2600 and 3200 cases of leukemia are diagnosed annually in children, and it is the first cause of death in children from 5 to 14 years (Jiménez‐Morales et al., 2017), this high prevalence is associated with different genetic variations (Jiménez‐Morales et al., 2008). Although there are no studies that have determined the prevalence of ALL in Cora and Huichol populations, Igwe et al., (2017) determined a prevalence of 34.4% of ALL in Hispanic population and 1% in native American population. Furthermore, Lepe‐Zúñiga et al., (2017) reported that ALL was the most frequent acute leukemia (75.3%), in a population from Chiapas. This study has great relevance since Chiapas is a state from Mexico with a large population of isolated ethnic groups with high level of consanguinity. Like the ethnic groups from Chiapas, Huichol population has a high degree of isolation, in addition to being a small population, so the genetic alterations could be more evident (Moreno‐Estrada et al., 2014).

*IKZF1* is a relevant gene of which some polymorphisms have been associated with the risk of developing BCP‐ALL in European, American, Afro‐American, and Hispano‐American populations. Considering that Mexico is a mestizo country but also possesses a well‐defined and unmixed indigenous population characterized by small effective population sizes under a model with a strong bottleneck (Moreno‐Estrada et al., 2014), it is important to know the specific risk that both polymorphisms represent for every ethnicity. A case‐control study with Brazilian population showed that homozygous variant of rs11978267 and rs4132601, especially, individuals with rs11978267 variant genotype had a higher risk of developing BCP‐ALL with IKZF1 haploinsufficiency in blast cells (Lopes et al., 2017). Górniak et al. (2014) determined the relation between polymorphic site rs4132601 and clinical features of pediatric patients with newly diagnosed ALL. They found that the GG genotype in recessive inheritance model at rs4132601 developed disease earlier in comparison with GT and TT genotypes.

The rs4132601 and rs11978267 are not located on the promoter or enhancer region of *IKZF1*. Some hypotheses about the role of these SNPs in ALL development have been proposed. One proposed explanation is that they could modify the expression of *FIGNL1*, due to these SNPs have been described as expression quantitative trait loci (eQTL), which are genomic loci that regulate the levels expression of the mRNA of the neighbor gene *FIGNL1*, an important gene involved in maintenance of genomic stability, in DNA double‐strand break (DBS) repair, via homologous recombination and cancer prevention. Likewise, this gene regulates the osteoblast proliferation and differentiation, and recently, has been associated with BCP‐ALL (Jayaram Laurynenka et al., 2020; Vijayakrishnan et al., 2018). Another hypothesis is that they could indirectly modify the gene expression when both polymorphisms are in linkage disequilibrium, as was previously described by Lopes et al., (2017), who suggest that haplotypes carrying variant genotypes of rs4132601 and rs11978267 may be related with leukemic transformation such as when *IKZF1* deletions are present.

The frequencies of the allele G of both polymorphisms were higher in Huichol than in Cora and Mestizo populations from Nayarit (Table 3). These results are very interesting, not only because the frequency of allele G in the Huichol population was higher than those in the Cora and Mestizo populations (Table 4), but also because they were higher than the frequencies reported in other populations of the world (TopMed‐study, 2019a, 2019b), and the sample analyzed in the present study represents almost the 1% of the total Huichol population from Nayarit. As we mentioned before, the presence of these polymorphisms increases the risk of ALL in some populations, but since the frequency of the risk allele is so high in the Huichol population, it is possible that individuals present a significant increase in the risk of developing ALL. As was mentioned before, both polymorphisms are in linkage disequilibrium, suggesting that they could be related to ALL risk; however, there is no data from the prevalence of ALL in indigenous Mexican population.

There are several reports describing the variability of genotypic and allelic frequencies of different kinds of polymorphisms of clinical and pharmacogenetic interest among indigenous Mexican groups, specifically the Huichol population (Gordillo‐Bastidas et al., 2010; Ramos, 2012; Wallander‐Compeán, 2011). These kinds of variations represent an increased risk of disease susceptibility; for instance, the presence in the Huichol population of the protective polymorphic alleles ADH1B*2 and ALDH2*2, which have been associated with a low capacity for alcohol consumption, is almost absent. In this study, the Huichol population presented the highest frequency of the allele CYP2E1*c2 reported to date (Gordillo‐Bastidas et al., 2010). Therefore, the Huichol population presented the highest allelic frequencies of CYP2E1*c2 (Gordillo‐Bastidas et al., 2010), rs4132601 G, and rs11978267 G reported to date (this work), and as we mentioned before, these three polymorphisms have been associated with an increase in the susceptibility to certain pathologies.

A few studies have determined the frequency of these polymorphisms in Mexican (HapMap, 2019; PAGE‐study, 2019) and Native American (PAGE‐study, 2019) populations. In the case of rs4132601, there were statically significant differences among the allelic frequencies reported in the Mexican population (PAGE‐study, 2019) compared with those found in our study for the Huichol population. In the case of the allelic frequencies in Mestizo and Cora populations obtained in the present study, there were no differences when compared with the allelic frequencies reported for a Mexican population (Table 6). Continuing with the analysis of this polymorphism, in the case of the allelic frequencies reported in a Native American population (PAGE‐study, 2019), there were also statically significant differences in comparison with a Huichol population but not with Mestizo and Cora populations. However, the authors do not specify the ethnic groups included in their study (PAGE‐study, 2019). Nevertheless, their results for allelic frequencies are very similar to those reported in other populations (PAGE‐study, 2019) (Table 4). Moreover, when the frequency of the rs11978267 was compared with the data previously reported for a Mexican population (HapMap, 2019), we found statistically significant differences compared with Mestizo, Cora, and Huichol populations (Table 6). These results could be because they reported genotypes AA = 0.68, AG = 0.32, and GG = 0, leading to a high allelic frequency of the ancestral allele of 0.84 (Table 6). They obtained these results from a Mexican population living in California, but the number of generations of ancestry of the subjects analyzed or their Mexican origins was not specified, and these could produce a bias in the frequency results.

The presence of the polymorphisms rs4132601 and rs11978267 has been associated with ALL development in several populations (Dai et al., 2014) but not in the Asian population (Bahari et al., 2016; Li et al., 2015; Xu et al., 2013). However, a larger prospective study is needed, which will allow us to determine whether or not in the Huichol population the presence of both polymorphisms increases the risk of developing ALL. In any event, there are no studies that support either of these two possibilities in this population, even, as mentioned above, there are no studies of prevalence of ALL in Huichol population.

The differences between Huicholes versus Meztizos and Coras can be attributed to they have a different origin. The Huicholes have been reported to be more related to the Tarahumara tribe in comparison with Coras and Mestizos (Páez‐Riberos et al., 2006). Moreover, the high prevalence of G alleles of both polymorphisms found in the present study is possibly the result of polygamy and the isolation of the Huichol population (Páez‐Riberos et al., 2006).

Moreno‐Estrada et al., (2014) reported that Huichol population has long homozygous tracts, on average over 10% of the genome in runs of homozygosity (ROH), in agreement with our results, where unexpected high homozygote frequencies of risk alleles in both variants (consistent with LD values), was found. Thus, the knowledge generated in the present work will be useful for carrying out association studies among these polymorphisms and indigenous Mexican subjects diagnosed with ALL.

In summary, the individuals analyzed in the present study represent the 1% of the total Huichol population from Nayarit, and presented the highest frequency of the risk allele reported so far in the whole world for both *rs*4132601 and *rs*11978267 polymorphisms, and also, they were in LD. It is necessary to pay attention to the results obtained, and from these, propose a strategy for monitoring the indigenous populations, which are considered the most vulnerable populations in Mexico with respect to access to specialized health systems, and thus they could receive early detection and medical attention and treatment for ALL.

## CONFLICT OF INTEREST

All authors declare that they have no conflicts of interest to disclose.

## AUTHORS’ CONTRIBUTIONS

Briefly, J.G.F. and M.F.A.P. performed the genotyping analysis and carried out the analysis of results, M.J.D.A. and N.V.P did the recruitment of the subjects, took the blood samples, and also carried out the analysis of results. D.E.S.J, D.G.B.A, and J.S.M. performed DNA extraction and Genotyping analysis. H.J.P.G., J.F.Z.Z., and J.M.A.C., participated in the analysis and interpretation of results. A.V.R. designed, conceptualized and supervised the study, was responsible for acquiring the funds and performed the genotyping analysis. All the authors did a literature review and contributed to writing and editing the manuscript and provided important comments.

## Data Availability

The data that support the findings of this study are openly available in ClinVar at https://www.ncbi.nlm.nih.gov/clinvar/. Accession number: SCV000993544 for rs4132601 and accession number: SCV000993545 for rs11978267.
